# The hypoglycemic effect of freeze‐dried fermented mulberry mixed with soybean on type 2 diabetes mellitus

**DOI:** 10.1002/fsn3.2321

**Published:** 2021-05-12

**Authors:** Xiao‐Shan Long, Sen‐Tai Liao, Er‐Na Li, Dao‐Rui Pang, Qian Li, Shu‐Cheng Liu, Teng‐Gen Hu, Yu‐Xiao Zou

**Affiliations:** ^1^ Guangdong Academy of Agricultural Sciences/Key Laboratory of Functional Foods Ministry of Agriculture and Rural Affairs/Guangdong Key Laboratory of Agricultural Products Processing Sericultural & Agri‐Food Research Institute Guangzhou China; ^2^ College of Food Science and Technology Key Laboratory of Advanced Processing of Aquatic Products of Guangdong Higher Education Institution Guangdong Provincial Key Laboratory of Aquatic Product Processing and Safety Guangdong Ocean University Zhanjiang China; ^3^ South China University of Technology/Guangdong Province Key Laboratory for Green Processing of Natural Products and Product Safety School of Food Science and Engineering Guangzhou China

**Keywords:** fermented mulberry, gut microbiota, hypoglycemic effect, probiotics, soybean, type 2 diabetes mellitus

## Abstract

Mulberry has significant hypoglycemic effect and can be used as an auxiliary food for people with type 2 diabetes. However, it is rich in carbohydrate and cannot be consumed directly by diabetic patients. In the study, we fermented the mulberry to reduce the content of glucose and fructose, and added the soybean to reduce the loss of probiotics during fermentation and then determined its hypoglycemic effect. We induced type 2 diabetes mellitus (T2DM) mice by streptozotocin and measured its blood glucose, serum biochemistry, hepatic and pancreatic histopathology, and the diversity of the gut microbiota. After 5 weeks of oral DFMS administration, the glucose tolerance was improved significantly in T2DM mice. Furthermore, there were also significant increases in superoxide dismutase activity and glutathione concentration, and marked reductions in the concentrations of malondialdehyde and free fatty acids. Moreover, DFMS also prevented histopathological changes and the increases in the activities of alanine transaminase and aspartate transaminase. DFMS treatment also markedly increased the richness of the gut microbial community. The abundance of *Bacteroidetes* was increased, and those of *Proteobacteria*, *Escherichia‐Shigella*, and *Lactobacillus* were reduced. In summary, DFMS has a clear hypoglycemic effect in mice with T2DM.

## Graphical abstract

In this passage, we produced a freeze‐dried preparation of fermented mulberry mixed with soybean (DFMS) to verify the protection of probiotic of soybean and determined its effect on hyperglycemia in diabetic mice. Our findings indicated that DFMS had an obvious hypoglycemic effect on T2DM mice, which is possibly related to its low carbohydrate content and the high levels of 1‐deoxyricogen and probiotic.

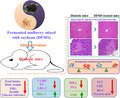

## 1 INTRODUCTION

Type 2 diabetes mellitus (T2DM) is a metabolic disease that is characterized by hyperglycemia and a series of metabolic disorders caused by insulin resistance. Chronic high blood glucose concentrations can induce excess free radical production, oxidative stress, and damage to islet *β* cells, which is followed by a reduction in insulin sensitivity (Hebi et al., 2017; Zhang et al., 2017). T2DM is also associated with abnormal glucose and lipid metabolism, which can be associated with disease in other organs and metabolic syndrome. Additionally, it also involves gut microbial imbalance, with changes in the diversity of the gut microbiota, including reduction in the abundance of *Bifidobacteria* and *Lactobacilli* and an increase in the abundance of *Sclerenchyma*, which may at least in part mediate the *β* cell damage and insulin resistance (Clara et al., 2004; Vionnet et al., 2000). More specifically, the imbalance of gut microbiota will change the function and metabolism of intestinal barrier, leading to the advancement of insulin resistance in T2DM, which results in the disorder of blood glucose and blood lipid metabolism (Arora et al., 2021). On the other side, gut microbiota also can treat T2DM mice via improving the insulin sensitivity, and then regulate glucolipid metabolism in body, and ameliorate the status of hyperglycemia, including the level of blood glucose and lipid, and other serum indexes (Karlsson et al., 2013; Lin et al., 2014). Sreng et al. have found that the glucose metabolism is associated with a specific change in gut microbiota ecology, which the increase of *Bifidobacterium* improves the glycemic control through insulin resistance (Sreng et al., 2019). Therefore, gut microbiota is closely related to diabetes and has interaction with each other. T2DM can cause the imbalance of gut microbiota, leading to the enhancement of insulin resistance and dysbiosis of glycemic status. On the contrary, gut microbiota plays a great role in T2DM which is able to advance the insulin sensitivity and then modify glycemic status in T2DM mice.

Currently, drugs are principally administered to reduce blood glucose and improve the anti‐oxidative capacity of patients with diabetes (Standl et al., 2018; Wang, Wang, et al., 2018; Wang, Zhao, et al., 2018; Wu et al., 2017). However, the long‐term use of some drugs may be harmful and cannot prevent further beta‐cell necrosis, while some oral hypoglycemic drugs are not effective in many patients. Therefore, it is necessary to develop alternatives. It has been reported that mulberry, soybean, grape seed, dandelion, persimmon leaf, ginkgo leaf, balsam pear, and other plant extracts are nontoxic and have positive effects in patients with T2DM (Brito et al., 2020; Chen et al., 2020; Li et al., 2019; Lim et al., 2012).

Mulberry is used as a traditional Chinese medicine and was recently stated to be the best health fruit in the list of foods and medicines published by the Ministry of Health of China, because it is rich in nutrients, containing fiber, amino acids, vitamins, and minerals; but it also contains a number of bioactive components, including alkaloids, anthocyanin, polyphenols, flavonoids, and polysaccharides, which have been shown to have antioxidant, hypoglycemic, and hypolipidemic effects (Chen et al., 2016; Wang, Cheng, et al., 2019; Wang, Shang, et al., 2019; Xiao et al., 2015). However, it contains relatively high levels of fructose and glucose, which are not suitable for excess consumption by diabetic patients. It was proved that the microorganisms could reduce the carbohydrate content of mulberry (Hanstock et al., 2004; Li et al., 2017; Wang et al., 2010). Li et al. studied the sugar‐reducing rate of different microorganisms of mulberry, and the result verified that the degradation rate of fructose and glucose was 86.49% and 66.12% respectively after fermentation by *lactobacillus* (Li et al., 2017). In our previous study (Long et al., 2020), we have confirmed that the fermented mulberry plays the better hypoglycemic effect than the unfermented mulberry in T2DM mice, which may be related to the change of bioactive substance content. After mulberry fermentation, the content of anthocyanins decreased, the content of 1‐deoxyricogen increased significantly, and the content of polyphenols and flavonoids remained unchanged. In addition, the nutrient and chemical compound of fermented mulberry were presented in Table 1, and fermented mulberry contains 3.8–18.54 mg/g protein, 21.2–30.46 mg/g total polyphenols, 0.3–6.65 mg/g total flavonoids, 0.67–2.23 mg/g 1‐deoxyricogen, and 0.5–2.86 mg/g anthocyanin. As we know that these nutrients and bioactive compounds of fermented mulberry play a central role in hypoglycemic activity in diabetic mice, especially the 1‐deoxyricogen, which has the ability to inhibit the metabolic effect of carbohydrates, repair pancreatic cells and then improve insulin resistance (Li et al., 2019; Nea & Ed, 2019), thus the higher 1‐deoxyricogen of fermented mulberry showed the better hypoglycemic effect. Moreover, 1‐deoxyricogen of mulberry exhibits gender‐specific modulating effects on hypercholesteremia and gut microbiota, which has significantly decreased the relative abundance of *Fimicutes,* and increased the abundance of *Akkermansia,* a beneficial genus with obesity and diabetes (Hou et al., 2020; Li et al., 2019). However, the probiotics of fermented mulberry were reduced significantly during the freeze‐drying that it is necessary to find the method to protect probiotic of fermented mulberry.

**TABLE 1:** The chemical composition of fermented mulberry and soybean

Compound		Content (mg/g) (dry weight)	
		Fermented mulberry (Jin et al., 2017; Khalifa et al., 2018; Li et al., 2019; Long et al., 2020; Mahboubi, 2019)	Soybean (Graça et al., 2016; Jeon & Park, 2012; Liang et al., 2006; Maleni et al., 2008; Zhang et al., 2018)
Nutrients	Protein	3.8–18.54	328–392
	Carbohydrates	0–11.2	187–253
	Fat	1.4–5.0	184–217
Bioactives	Total polyphenols	21.2–30.46	1.89–3.84
	Total flavonoids	0.3–6.65	0.33–0.96
	isoflavones	–	0.4–5.0
	1‐deoxyricogen	0.67–2.23	–
	anthocyanin	0.5–2.86	1.30–3.55

Probiotic is a kind of active microorganism which is beneficial to the host, and it is widely used as functional food and nutraceuticals. Interestingly, probiotic has the ability to adapt to the human intestinal environment, improve the intestinal micro‐ecosystem, regulate the intestinal flora, and then ameliorate the glycolipid metabolism of the human body (Barros et al., 2020; Tonucci et al., 2015). In addition, metabolic disorders such as T2DM can be treated by probiotics, such as Bifidobacterium and Lactobacillus, which are the live and good bacteria and keep our gut healthy (Arora et al., 2021). For example, Lactobacillus can regulate the blood glucose level of host by inhibiting the activity of adrenal sympathetic nerve, stimulating the activity of gastric vagus nerve, and reducing the secretion of adrenaline (Hsieh et al., 2013; Tonucci et al., 2015). Some research illustrated that lactobacillus can adhere to intestinal mucosa through adhesins on their own surface and covalently combine with intestinal mucosal cell receptor proteins to form a biological barrier and release immune factors, thus enhancing the immune function of the body (Ghadimi et al., 2008; Lin et al., 2014). Moreover, the intake of *Lactobacillus* is able to inhibit the activity of *α*‐glucosidase, reduce insulin resistance, repair the oxidative damage of islet *β* cells, and improve insulin sensitivity (Ejtahed et al., 2011; Maria et al., 2016). Probiotic therapy has a good application prospect in disease prevention and reconstruction of intestinal microecology health due to its low cost, high safety and high reliability, and has gradually become a research hotspot of diabetes prevention and treatment.

Based on previous research in our laboratory, soybean has the ability to protect the survival rate of probiotic during the freeze‐drying process. The possible protection mechanism is that soybean contains soluble fiber, isoflavones, sterols, oligosaccharides, saponins, phospholipids, and other active ingredients, which are able to embedding probiotics during the freeze‐drying process. Furthermore, some reports state soybean also possesses the effects of hypoglycemic, antioxidant, and anti‐inflammatory (Ali et al., 2004; Bai et al., 2020; Ji et al., 2011; Yuan et al., 2012). Lim et al. proved that the extracts of soybean could inhibit hyperglycemia and free radical‐mediated oxidative stress, prevent the functional changes in vascular reactivity in STZ‐induced diabetes (Lim et al., 2012). In fact, the hypoglycemic activity and protection to probiotic of soybean may be attributed to their active components. As shown in Table 1, soybean contains 328–392 mg/g protein, 1.89–3.84 mg/g total polyphenols, 0.33–0.96 mg/g total flavonoids, 0.4–5.0 mg/g isoflavones, and 1.30–3.55 mg/g anthocyanin. Therefore, in the present study, we produced a freeze‐dried preparation of fermented mulberry mixed with soybean (DFMS) to verify the protection of probiotic of soybean and determined its effect on hyperglycemia in T2DM mice.

## 2 MATERIALS AND METHODS

### 2.1 Chemicals

Streptozotocin (STZ) was purchased from Sigma (St. Louis, MO, USA). Assay kits were provided by Nanjing Jiancheng Bioengineering Institute (Nanjing, China).

### 2.2 Materials

Fresh mulberry fruit (*Morus alba* Linn.), harvested from the Huadu Bosun field production base of Guangdong Academy of Agricultural Science, was grown to ~90%‐maturity and identified as the Da 10 variety. Fresh fruit, free of contaminants (lime and sand), disease, and insects were picked. Soybeans were purchased from Heilongjiang Province North Pure Agricultural Products Development Company Ltd. (China). *Leuconstoc mesenteroides* and *Saccharomycetes* were provided by the Strain Preservation Center of the Sericultural & Agri‐Food Research Institute, Guangdong Academy of Agricultural Sciences (Guangzhou, China). The *Leuconstoc mesenteroides* and *Saccharomycetes* were both provided by the Strain Preservation Center of the Sericultural and Agri‐Food Research Institute, Guangdong Academy of Agricultural Sciences.

### 2.3 Sample preparation

To produce DFMS, mulberry powder and distilled water were mixed at a ratio of 1:4, fermented by *Leuconstoc mesenteroides* for 96 hr and *Saccharomycetes* for 6 hr. After fermentation, the soybean was beaten into sirup and added to the fermented mulberry liquid and stirred evenly, and then freeze‐dried. The mass ratio of soybean sirup to the fermentation liquid was 1:5, according to our laboratory's previous research (Li et al., 2017).

### 2.4 Determination of monosaccharide content

Monosaccharide content was determined using high‐performance liquid chromatography according to the method reported by Zheng et al. (2014), with a slight modification. The analysis was carried out on an evaporative light scattering detector with a drift tube temperature of 45°C, using a Shodex Asahipak NH2P‐504E column (4.6 mm × 250 mm, 5 μm) at 30°C for 15 min. The monosaccharides were eluted using an acetonitrile (75% v/v) mobile phase at a flow rate of 1 ml/min.

### 2.5 Determination of *Leuconstoc mesenteroides* and *Saccharomycetes* content

The total number of *Leuconstoc mesenteroides* and *Saccharomycetes* was determined by dilution plate coating method in GB4789.2‐2016.

### 2.6 Animals

Male Kunming mice (20 ± 2 g) were purchased from the Experimental Animal Center of the Guangzhou University of Chinese Medicine [SYXK (yue) 2015‐0149]. The source number of the mice was NO.44005800005790, and the inspection date was July 7th, 2017. The study was approved by the Experimental Animal Management Committee of the Sericultural & Agri‐Food Research Institute, Guangdong Academy of Agricultural Sciences.

### 2.7 Induction of T2DM in mice and administration of test substances

The mice were maintained in laminar flow cabinets under specific pathogen‐free (SPF) conditions at 23 ± 3℃ and 55 ± 15% humidity, with free access to sterile water and regular chow diet. After 7 days of acclimation, we randomly selected 12 mice to form a control group (NC group) and induced diabetes in the other mice by injecting 100 mg/kg STZ (Dong et al., 2020). One week later, the blood glucose of most mice increased, and the mice with unchanged blood glucose were injected intraperitoneally with 30 mg/kg STZ again. As we know that, STZ belongs to glucosamine nitrosourea, which is a DNA alkylation reagent. It can enter cells alone through glucose transporter 2 (GLUT2). Moreover, it can selectively destroy islet *β* cells in mice or rat, and induce type 1 or 2 diabetes and then lead to blood glucose imbalance in mice or rat, and the degree of *β* cell damage determines the type of diabetes (Dong et al., 2020). The fasting blood glucose (FBG) concentrations of these mice were subsequently measured, and those with concentrations ≥7 mmol/L were defined as having diabetes (Andallu et al., 2012; Yang et al., 2015). The diabetic mice were randomly allocated to four groups: a diabetic model group (DM group, *n* = 12), a positive control group (PC group, *n* = 12, which were administered acarbose), and a DFMS‐treated group (*n* = 12). According to our previous study, the dose of DFMS administered by gavage was 2.26 g/(kg·bw)/day, equivalent to mulberry dose of 230 mg/(kg·bw)/day (mulberry was the main material in DFMS) (Long et al., 2020). All the groups underwent gavage once daily for 5 weeks. Food intake was recorded every 3 days, body weight was recorded every 5 days, and FBG was measured every 7 days during this period. After 5 weeks, the mice were terminally anesthetized by intraperitoneal injection of 1% pentobarbital sodium (40 mg/kg), then blood was collected from the heart and the organs of interest were harvested.

### 2.8 Oral glucose tolerance testing (OGTT)

At the end of the 5th week of the study, and after an overnight fast, 2 g/kg glucose was administered orally to all the mice and blood samples were collected from each 0, 30, 60, and 120 min afterward for the measurement of blood glucose using glucose meter (Zhi et al., 2012). The areas under the blood glucose curve (AUC) were calculated using Prism 7 (GraphPad, San Diego, CA, USA).

### 2.9 Biochemical analysis

The liver, heart, kidney, and spleen were quickly removed from the mice and weighed. The collected blood was centrifuged at 4℃ and 3,000 rpm for 15 min, and then the serum was removed and stored at −80℃. The serum triglyceride (TG), total cholesterol (T‐CHO), low‐density lipoprotein‐cholesterol (LDL‐C), high‐density lipoprotein‐cholesterol (HDL‐C), glutathione (GSH), malondialdehyde (MDA), free fatty acid (FFA), and creatinine (CRE) concentrations; the blood urea nitrogen (BUN); the superoxide dismutase (SOD), alanine transaminase (ALT), aspartate transaminase (AST) activities, and the liver glycogen content, hexokinase (HK) activity, C‐peptide and insulin were measured according to the kit instructions. HOMA‐IR was calculated by fasting blood glucose (FBG) and fasting insulin (HOMA‐IR = FBG (mmol/L) × insulin (mIU/L)/22.5).

### 2.10 Histopathology of the liver and pancreas

Pancreas and liver samples were rinsed with precooled PBS and fixed in 10% formalin. After 24 hr, they were embedded in paraffin and 5‐μm‐thick sections were prepared and stained with hematoxylin and eosin (H&E), mounted, and examined using a light microscope.

### 2.11 MiSeq genome sequencing analysis of microbial community structure

High‐throughput sequencing of the V3–V4 regions of the fecal microorganismal 16S rRNA genes was performed. Standardization, pooling, and sequencing were performed on the amplified DNA, and the sequences were screened, including by hybridization, clustering, and chimera detection. Sequences with a similarity ≥97% were defined as operational taxonomic units (out). The representative sequence of OTU was used for species identification. Then, multiplex clustering and mass filtration, OTU selection, taxonomic distribution, phylogenetic reconstruction, and diversity analysis were used to study the diversity of the microbial community (Jie et al., 2020; Jin et al., 2019).

### 2.12 Statistical analysis

The data are expressed as mean ± *SE*. Duncan's post hoc test was used to test the mean differences between groups. *p* < .05 was considered to represent statistical significance.

## 3 RESULTS AND DISCUSSION

### 3.1 The content of monosaccharide and probiotics of DFMS

The content of glucose and fructose in mulberry before fermentation was 526.41 mg/g and 10.09 mg/g (dry weight), respectively, while DFMS was 12.56 mg/g and 0.29 mg/g (dry weight), respectively. The result indicated that the content of monosaccharide in mulberry powder after freeze‐drying was significantly reduced, and DFMS was a kind of low‐sugar product. After fermentation, the content of *Leuconstoc mesenteroides* and *Saccharomycetes* in mulberry was severally 7.18 log (cfu/ml) and 6.91 log (cfu/ml), DFMS was severally 6.59 log (cfu/ml) and 6.340 log (cfu/ml), and the above two kinds of bacteria retention rate were as high as 91.85% and 91.75% respectively. The result showed that soybean could protect probiotics in the lyophilization of mulberry fermentation liquid.

### 3.2 Effect of DFMS on the food intake, body mass, FBG, organ masses, and glucose tolerance of the mice

**FIGURE 1:**
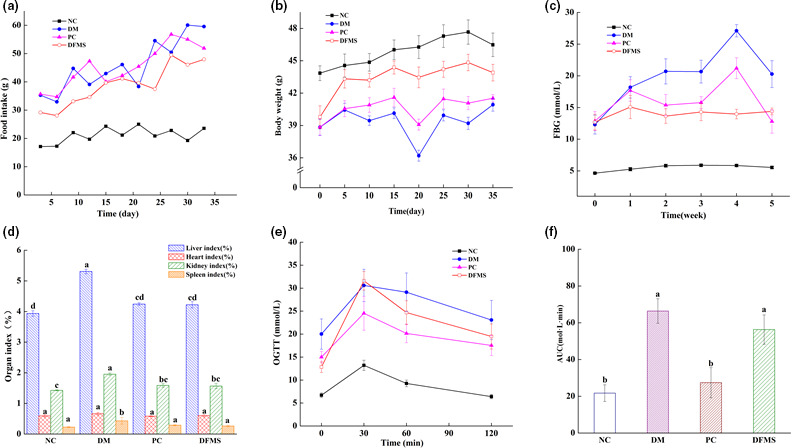
Effect of DFMS administration on body intake, body mass, FBG, organ masses, and glucose tolerance in diabetic mice

As shown in Figure 1a, the food intake of the DM group was significantly higher than that of the NC group (*p* < .05). At the end of the study, the food intakes of the PC and DFMS groups were significantly lower than that of the DM group (*p* < .05). Diabetic mice that were administered DFMS or acarbose had a higher body weight than that of the DM group (Figure 1b, *p* < .05), and this was especially true of the DFMS group. Furthermore, the FBG of the DM group was much higher than that of the NC group (Figure 1c). Conversely, the DFMS and PC groups showed significantly lower FBG than the DM group (*p* < .05). Notably, the DFMS group had the strongest effect to ameliorate hyperglycemia among the experimental groups. Compared with the NC group, the liver, kidney, and spleen masses in the DM control group were significantly higher (Figure 1d, *p* < .05). Conversely, there were no differences among the PC, DFMS, and NC groups (Figure 1d, *p* > .05). These results indicated that the diabetes damaged the liver, kidney, and spleen of mice, and that this was ameliorated by DFMS administration.

OGTT was conducted at the end of the study. The blood glucose concentrations of the experimental mice increased when they were orally administered glucose and peaked 30 min later (Figure 1e), then returned to the initial concentrations over 120 min in most of the groups. However, that of the DM group remained high for the whole 120 min (Figure 1e). The AUC calculated over the 120 min was significantly lower in the DFMS and PC groups than in the DM group (Figure 1f, *p* < .05). These results indicated that DFMS had a positive effect on glucose tolerance in diabetic mice. In summary, there were significant decreases in the blood glucose and food intake, and increases in body mass and glucose tolerance in the DFMS‐treated group versus the DM group (*p* < .05). Thus, DFMS has beneficial effects in diabetic mice.

### 3.3 Effect of DFMS on serum lipid profile

**FIGURE 2:**
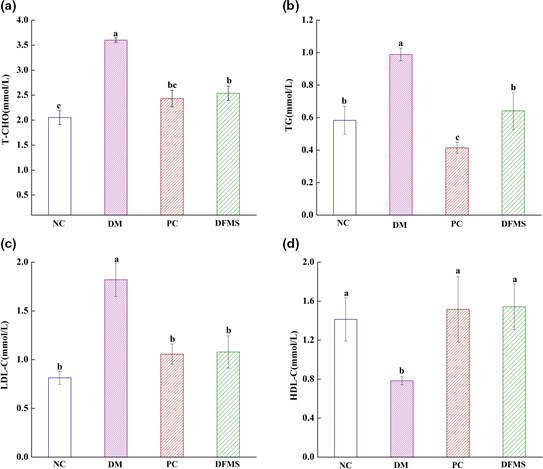
Effect of DFMS on serum lipid concentration

The serum concentrations of T‐CHO, TG, LDL‐C, and HDL‐C in the mice are shown in Figure 2. These are essential for whole‐body metabolism (Wang, Wang, et al., 2018; Wang, Zhao, et al., 2018). Mice in the DM group had higher T‐CHO, TG, and LDL‐C concentrations and a lower HDL‐C concentration than those of the NC group (*p* < .05), which confirms that the diabetes was associated with a blood lipid disorder. Treatment of the diabetic mice with DFMS for 5 weeks was near‐normalized these lipid concentrations.

Glycolipid metabolism disorder, including blood glucose and lipid metabolism, is one of the main metabolic disorders in T2DM, which is mainly due to the diversity of the gut microbiota, the function of islet *β* cells, and the obstruction of glucose transport (Dong et al., 2020). The imbalance of gut microbiota will change the function and metabolism of intestinal barrier, leading to the advancement of insulin resistance in T2DM, which results in the disorder of blood glucose and lipid levels (Arora et al., 2021; Karlsson et al., 2013). We hypothesize that the mechanism for this improvement in blood lipid concentrations may be that DFMS alters the diversity of gut microbiota in mice, promoting its insulin sensitivity, which has the effect of inhibiting fat synthesis, and increasing fat metabolism, cholesterol metabolism, and bile acid excretion (Hernández‐Quiroz et al., 2019).

### 3.4 Effect of DFMS on oxidation and anti‐oxidative parameters in diabetic mice

Free radicals are by‐products of metabolism that are highly toxic in high concentrations. They can cause the oxidation of amino acids, sugars, proteins, nucleic acids, and other compounds in various tissues, leading to oxidative stress and inflammation, and thus organ damage (Song et al., 2021; Wang, Wang, et al., 2018; Wang, Zhao, et al., 2018).

It has been shown that SOD activity and the concentrations of MDA, GSH, and FFA reflect the production of oxygen free radicals and the degree of oxidative damage in the body, and thereby reflect antioxidant capacity (Dang et al., 2018; Mi et al., 2016). SOD is an enzyme that eliminates harmful substances produced during metabolism and GSH can transform toxic substances into nontoxic substances and help remove them from the body (Dang et al., 2018). Both SOD and GSH effectively remove oxygen free radicals and reflect the antioxidant capacity of the body. Conversely, MDA and FFA are produced during lipid and glucose metabolism and may induce oxidative stress when present in excess (Mi et al., 2016).

**FIGURE 3:**
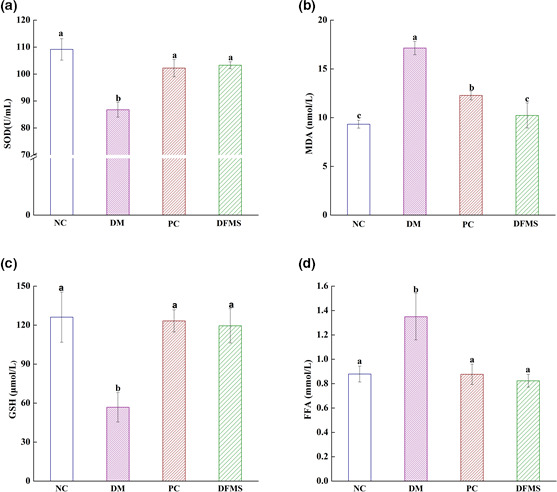
Effect of DFMS on serum SOD activity and MDA, GSH, and FFA concentration

To determine the oxidative status of each group of mice, we measured their serum SOD activity and MDA, GSH, and FFA concentrations. As shown in Figure 3, compared with the NC group, the level of SOD, GSH in the serum of DM group was observably declined by 86.77 U/mL, 56.82 μmol/L, respectively (*p* < .05); and the concentrations of MDA and FFA were 17.14 mmol/L and 1.35 mmol/L, respectively, (*p* < .05). However, DFMS treatment near‐normalized all four indices in the study (Figure 3a–d, *p* < .05).

### 3.5 Effect of DFMS on indices of liver, heart, and kidney pathology and function

**FIGURE 4:**
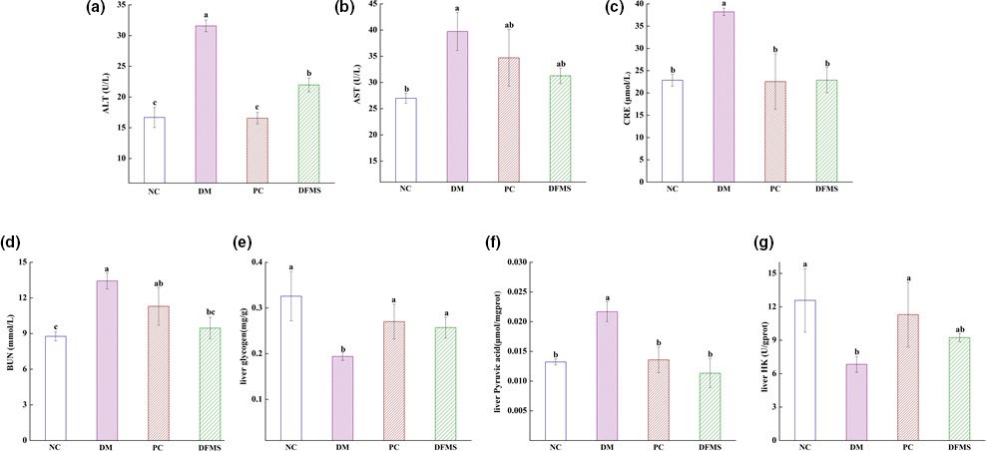
Effect of DFMS on serum ALT and AST activities, BUN, serum CRE concentration, liver glycogen and pyruvic acid content, and liver HK activity

ALT is a sensitive marker of acute hepatocyte damage. AST is mainly expressed in the myocardium, but also in the liver, skeletal muscle, and kidney, and is used as an auxiliary marker of myocardial infarction, myocarditis, and liver damage. It has been reported that diabetes increases the serum activities of ALT and AST, reflecting damage to the liver and heart (Irani et al., 2018). As shown in Figure 4a, the serum ALT activity was lower in the DFMS group than in the DM group (21.98 U/L vs. 31.57 U/L, respectively; *p* < .05), such that there was no significant difference in the serum AST activity between the DFMS and NC groups (31.27 U/L vs. 26.99 U/L, respectively; *p* > .05; Figure 4b). These results suggest that DFMS reduces the liver injury associated with diabetes.

CRE and BUN in the blood are excreted through the kidneys; therefore, their circulating concentrations increase when renal function is impaired and they can be used as indicators of kidney function (Padee et al., 2010). As shown in Figure 4c,d, we found that the serum CRE and BUN in the DM group were higher than those of the NC group (DM group: 38.21 and 13.43 μmol/L; NC group: 22.86 and 8.77 μmol/L, respectively), but both were reduced by the administration of DFMS to the diabetic mice (*p* < .05).

Glucose is stored in the liver in the form of glycogen, which can be broken down to liberate glucose to supply energy when required (Wang et al., 2017). Pyruvate is a product of glycolysis that can enter the tricarboxylic acid cycle to complete the aerobic oxidation of glucose, and is also an intermediate in the mutual transformation of sugar, fat, and amino acid (Zhao et al., 2017). HK is a key kinase that is widely expressed and inhibited by glucose‐6‐phosphate and ADP. Diabetes is characterized by low liver glycogen storage, an inhibition of glycolysis, and a higher rate of gluconeogenesis, which are associated with high HK activity and pyruvate concentration (Jimenez‐Chillaron et al., 2005). Therefore, liver glycogen and pyruvate content and HK activity reflect diabetic status. Figure 4e,g show higher glycogen content and HK activity in the liver of the DFMS and PC groups than in the DM group (*p* < .05), such that there were no significant differences in these parameters between the NC and DFMS groups (*p* > .05). Furthermore, the pyruvate content of the liver was significantly lower in the DFMS and PC groups than in the DM group (Figure 4f, *p* < .05). These results suggest that DFMS treatment may ameliorate defects in liver, heart, and kidney function.

### 3.6 Effect of DFMS on islet *β* cells

As we know that, C‐peptide and insulin are the secretion products of islet *β* cells, which can reflect the function of islet *β* cells and have great significance in the diagnosis and treatment of diabetes, and adiponectin is an insulin sensitizer, which can improve insulin resistance in mice (Hernández‐Quiroz et al., 2019; Maria et al., 2016). Therefore, we measured the serum C‐peptide, insulin and adiponectin, and calculated the index of HOMA‐IR by FBG and insulin, so as to study the effect of DFMS on islet *β* cells in T2DM mice.

**FIGURE 5:**
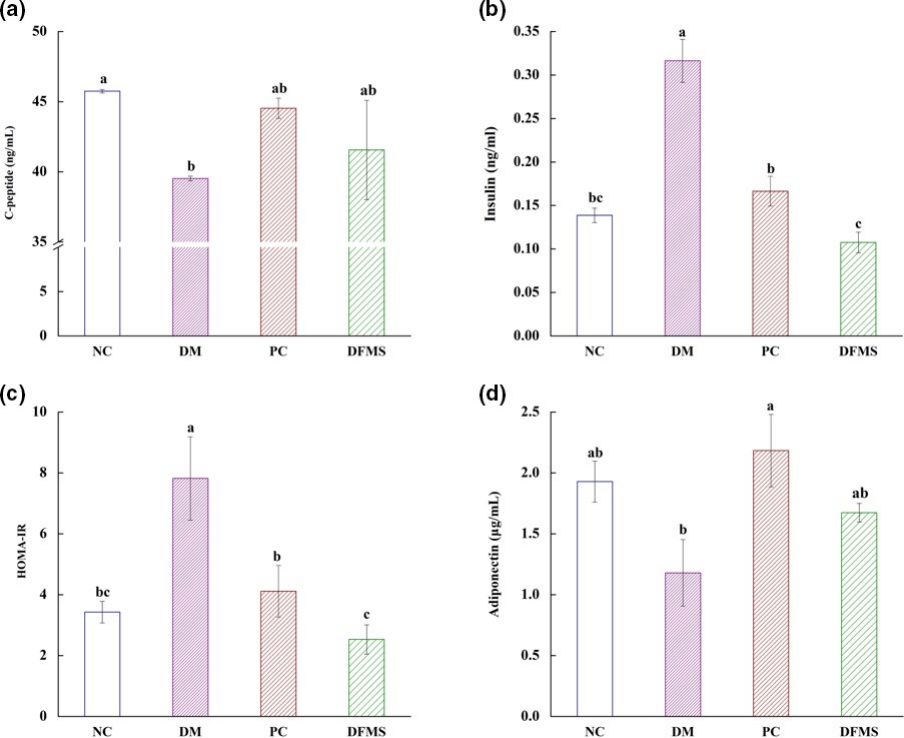
Effect of DFMS on the serum C‐peptide, insulin, HOMA‐IR and adiponectin

As shown in Figure 5a, the content of C‐peptide from NC, PC, and DFMS groups was higher than DM group, which proved that these groups increased the content of C‐peptide in T2DM mice to a certain extent. The content of insulin was shown in Figure 5b. We found that DFMS can significantly intensify the content of insulin in T2DM mice (*p* < .05). The index of HOMA‐IR was calculated by FBG and insulin, the result was presented in Figure 5c, and the NC, PC, and DFMS groups had an obvious reduction, comparing with DM group (*p* < .05). Both the result of insulin and HOMA‐IR proved that DFMS markedly decreased the insulin resistance of islet *β* cells in T2DM mice. In addition, we found that the serum adiponectin in the DM group was lower than those of the experiment groups (DM group: 1.18 μg/mL; NC group: 1.93 μg/mL, PC group: 2.18 μg/mL, DFMS group: 1.67 μg/mL, respectively). All of the results of these indexes illustrated that DFMS may repair the damaged islet *β* cells, improve the insulin resistance, and then ameliorate the insulin sensitivity in T2DM mice.

### 3.7 Effect of DFMS on hepatic and pancreatic histopathology

**FIGURE 6:**
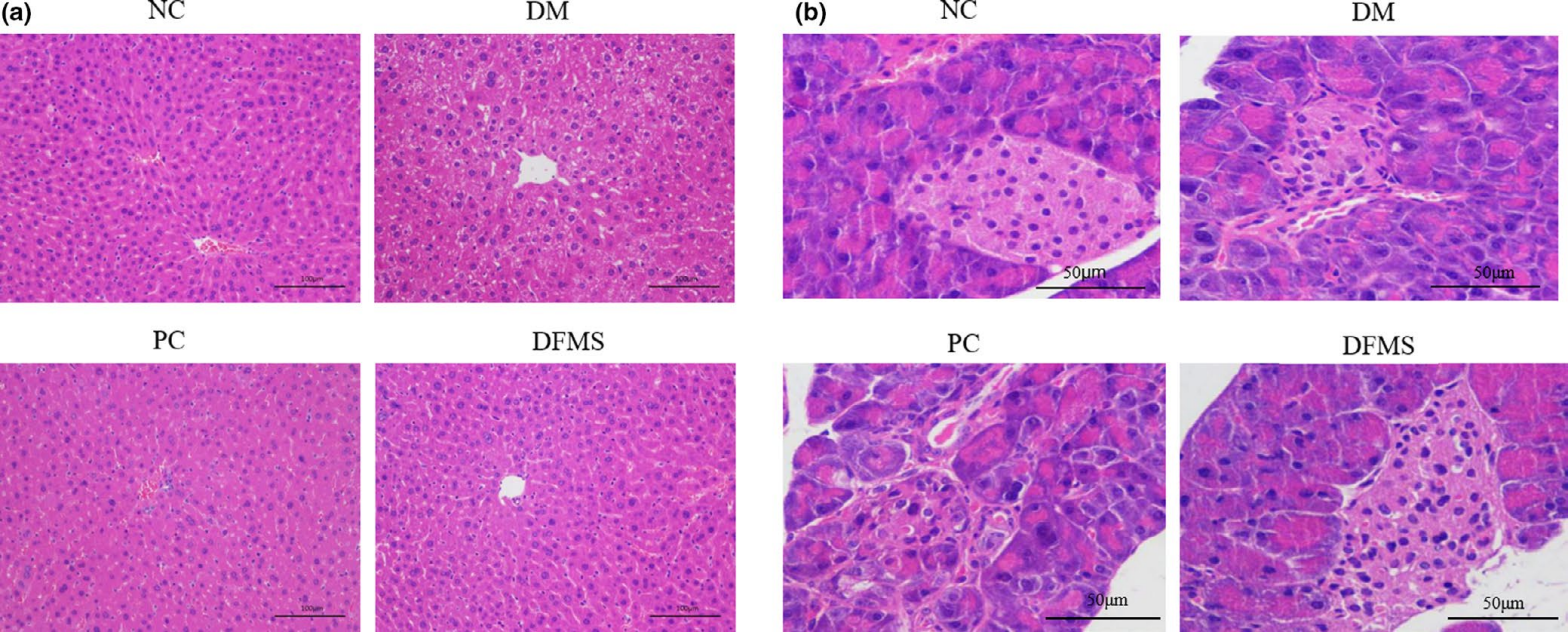
Effect of DFMS on the histopathology of diabetic mice (a) Histopathology of the liver (H&E staining, scale bar: 100 μm). (b) Histopathology of the pancreas (H&E staining, scale bar: 50 μm)

The histology of the liver and pancreas of the mice was examined following H&E staining of paraffin sections (Figure 6). The hepatocytes of the NC group were neatly arranged, well defined, and round or oval in shape (Figure 6a). Conversely, those of the DM group were disorganized, less densely packed, unequally sized, and contained cytoplasmic droplets. However, compared with the DM group, the hepatocytes of the DFMS group were neatly arranged, more densely packed, and contained fewer cytoplasmic droplets (Figure 6b).

The islet cells of the NC group were present in large numbers, arranged regularly, and showed normal morphology, with regular, clear cell boundaries, and normal cytoplasm. In the DM group, the islet cells were irregular in structure, shape, and distribution, with abnormally shaped nuclei. Most of the cells in the DM group were significantly smaller and had indistinct boundaries. However, compared with the DM group, there were more islet cells in the DFMS group and their morphology was relatively normal, which indicates that DFMS prevents a most diabetes‐associated pathology.

These results demonstrate that DFMS prevents the development of pathology in liver and pancreatic cells, which is associated with improvements in liver and kidney function and whole‐body metabolic status. Our results are consistent with those of previous studies in which other potential diabetic therapies were tested. For example, Li *et al*. found that the hepatic and pancreatic histopathology of diabetic mice was ameliorated by 12 weeks of treatment with multi‐strain probiotics (Li et al., 2016).

### 3.8 Effect of DFMS on the diversity of the gut microbiota

The gut microbiota is the largest and most complex microbial community in the human body, but it is also referred to as an important metabolic “organ.” It plays an important role in the intake of nutrients, the growth and development of cells, and metabolism of the host (Rad et al., 2017), and accumulating data show that it may mediate some of the effects of anti‐diabetic therapies (Min et al., 2009; Zhao et al., 2016). For example, Hu *et al*. studied the hypoglycemic effect of 1‑deoxynojirimycin, isolated from mulberry leaves, and found that it promoted the growth of beneficial bacteria, such as lactic acid‐generating bacteria, and inhibited that of harmful bacteria, thereby improving the intestinal microbial composition and alleviating intestinal disorders in diabetic mice (Hu et al., 2019).

**FIGURE 7:**
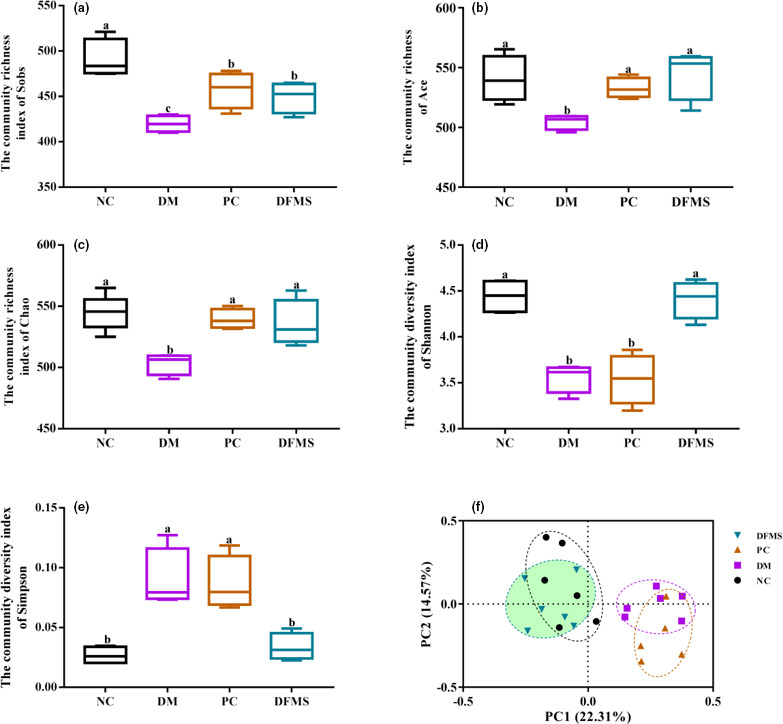
Effect of DFMS on the community diversity and structure of fecal microbiota. Alpha diversity, assessed using the (a) Sobs diversity index; (b) Acc diversity index; (c) Chao diversity index; (d) Shannon diversity index. (f) Principal co‐ordinate analysis, an index of the beta diversity of gut microbiome, was performed using Bray–Curtis metrics

In the present study, we performed a metagenomic analysis of the V3–V4 region of the 16S rDNA gene sequences to determine the effect of DFMS on the gut microbiota. Figure 7a–e show the alpha diversity of the microbial population at the OTU level. As shown in Figure 7a, the Sobs index of DFMS, which is positively correlated with community diversity, had significant difference compared with the model mice group (*p* < .05). Additionally, the Acc, Chao, and Shannon indices, which reflect the richness of the community, were much higher in the DFMS group than in the DM group (*p* < .05), such that they were similar to the values of the NC group (Figure 6b–d).

The Simpson index negatively correlates with community diversity. It was higher in the DM group than in the NC group (*p* < .05), but was similar in the DFMS and NC groups. The distance‐based Bray–Curtis method was used to conduct principal coordinate analysis to assess the similarity and distance of the ecological complexity among the experimental samples (beta‐diversity; Figure 7f). According to this index, the microbial composition of the DFMS and NC groups was similar, but highly dissimilar to that of the DM group (*p* < .05).

These findings show that diabetes is associated with changes in the structure and ecological diversity of the gut microbiota, with a higher abundance of harmful bacteria and a lower abundance of beneficial bacteria, and that the microbial populations are largely normalized by DFMS administration. In this research, the level of glucose and glucose tolerance was modified, and the lipid metabolism also was improved after treated by DFMS, which may relate to the diversity of the gut microbiota. The possible mechanism is that DFMS perfected the diversity of gut microbiota, thus improving the insulin sensitivity and metabolism of blood glucose and lipid. It was found that the specific change in gut microbiota ecology is associated with glucose metabolism which may ameliorate the insulin sensitivity, and then improve glucolipid metabolism in body, and ameliorate the status of hyperglycemia, including the level of blood glucose and lipid so as to play a good hypoglycemic effect (Karlsson et al., 2013; Lin et al., 2014; Nsab et al., 2019).

### 3.9 Effect of DFMS on gut microbial composition

**FIGURE 8:**
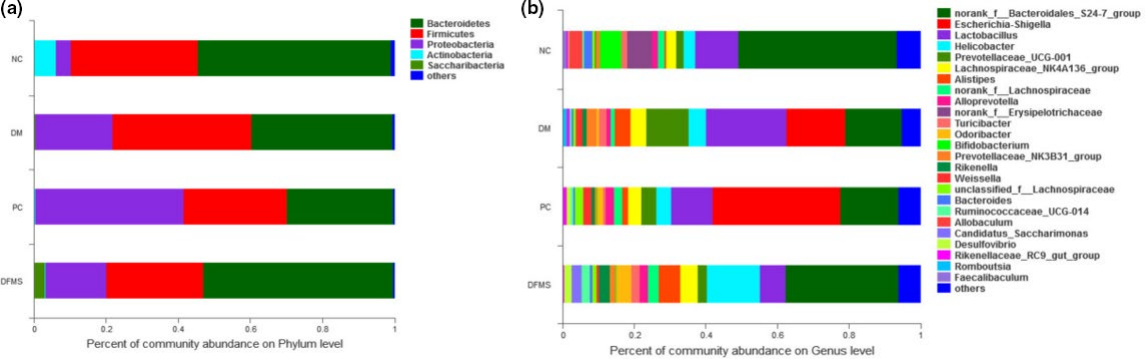
Effect of DFMS on the gut microbial composition

The microbiota helps the small intestine to absorb more glucose from food, accelerates glucose transport, generates short‐chain fatty acids, and regulates glucose and lipid metabolism in the host, and therefore, the composition of the gut microbiota plays an important role in diabetes and its treatment (Caterina et al., 2015). In the present study, we investigated the relationship between the composition of the gut microbiota and diabetes at the phylum and genus levels. At the phylum level, DFMS substantially increased the abundance of *Bacteroidetes* and reduced the abundance of *Proteobacteria* in the diabetic mice (*p* < .05). However, the abundances of both the *Escherichia‐Shigella* and *Lactobacillus* genera were lower in the DFMS group than in the DM group (Figure 8b, *p* < .05). Therefore, DFMS was reliable to regulate the abundance of gut microbiota. Li *et al* discovered that the 1‐deoxyricogen significantly declined the relative abundance of *Fimicutes*, which mainly attributed to the reduction of the genera *Turicibacter*, *Lactobacillus*, having gut microbiota‐modulating and glucose tolerance‐modulating effects (Li et al.,^2019^). It is consistent with our results that DFMS can modulate the abundance of gut microbiota, thus enhancing the advance of glucose status.

In short, the present study shows that DFMS played a positive effect in T2DM mice that could reduce blood sugar level, improve blood lipid and oxidative stress level, regulate abundance of gut microbial, which is possibly related to its low carbohydrate content and the high levels of 1‐deoxyricogen and probiotic. It has to be proved that 1‐deoxyricogen is able to suppress the *α*‐glucosidase, restraining the decomposition of disaccharides into glucose, and inhibit the metabolic effects of carbohydrates, optimize the enrichment and composition of gut microbiota, repair pancreatic cells, and then improve insulin resistance (Li et al., 2019; Nea & Ed, 2019). In addition, probiotic has the ability to adapt to the human intestinal environment, improve the intestinal micro‐ecosystem, regulate the abundance and composition of gut microbiota, and then ameliorate the glycolipid metabolism of the human body (Wang, Cheng, et al., 2019; Wang, Shang, et al., 2019). Hsieh *et al*. have proved that oral administration of *Lactobacillus* improves insulin resistance and ameliorates hepatic steatosis in high fructose‐fed rats (Hsieh et al., 2013). Moreover, the soluble fiber, isoflavones, and sterols in soybean are also known to be effective hypoglycemic substances, which maybe assist in lowering blood sugar. These substances may form a mucous membrane in the gastrointestinal tract, which slows down the digestion and absorption of food nutrients and limiting hyperglycemia, or they may stimulate the secretion of insulin by residual pancreatic beta‐cells (Fei et al., 2014; Slavin et al., 2009).

## 4 CONCLUSIONS

The DFMS, with low carbohydrate content and the high levels of probiotic, was successfully produced. DFMS administration reduced the food intake, blood glucose, and organ masses of T2DM mice, increased their glucose tolerance and antioxidant activity, and improved their blood lipid profile and anti‐oxidative activity. It also reduced damage to and might improve the function of the kidney, liver, and pancreas. Finally, the taxonomic diversity of the gut microbiota was improved by 5 weeks' treatment with DFMS. Thus, DFMS has various hypoglycemic effects in diabetic mice.

## ETHICAL APPROVAL

The authors declare that they do not have any conflict of interest. The study's protocols and procedures were ethically reviewed and approved by the Animal Care Committee at the Sericultural & Agri‐Food Research Institute, Guangdong Academy of Agricultural Sciences.

## Data Availability

The research data are not shared.
